# Prescription patterns of ambrisentan in some cities of Colombia

**DOI:** 10.1111/crj.13736

**Published:** 2024-03-19

**Authors:** Luis Fernando Valladales‐Restrepo, Andrés Gaviria‐Mendoza, Manuel Enrique Machado‐Duque, Álvaro Vallejos‐Narváez, Jorge Enrique Machado‐Alba

**Affiliations:** ^1^ Grupo de Investigación en Farmacoepidemiología y Farmacovigilancia Universidad Tecnológica de Pereira‐Audifarma S. A Pereira Risaralda Colombia; ^2^ Grupo de Investigación Biomedicina, Facultad de Medicina Fundación Universitaria Autónoma de las Américas Pereira Colombia; ^3^ Megalabs Colombia SAS Bogotá Colombia

**Keywords:** ambrisentan, Colombia, hypertension, medication adherence, pharmacoepidemiology, pulmonary

## Abstract

**Introduction:**

Ambrisentan is a selective type A endothelin receptor antagonist that has shown significant effectiveness and safety in the management of patients with pulmonary hypertension. Its use pattern with real‐world evidence in Colombia is unknown.

**Objective:**

The objective of this study is to determine the prescription patterns of ambrisentan in some cities of Colombia.

**Methods:**

A longitudinal descriptive study on the prescription patterns of ambrisentan in patients with pulmonary hypertension (all the groups) was conducted between January 2021 and December 2022 based on a population database of members of the Colombian Health System. Adherence at 1 year was determined using the Medication Possession Ratio (days the drug was dispensed/days from first dispensing to the end of the follow‐up period × 100). Descriptive analysis was carried out.

**Results:**

Sixty‐seven patients taking ambrisentan were identified in 10 cities of the country. The individuals had a median age of 51.5 years (interquartile range‐IQR: 39.8–64.0 years), and 82.1% were women. The drug possession rate was 82.2% (IQR: 65.0–96.8%), and persistence at 1 year was present in 49.3% (*n* = 33) of the cases. The average dose was 8.8 ± 5.0 mg/day, and 76.1% (*n* = 51) received it in combination therapy, mainly with phosphodiesterase type 5 inhibitors (61.2%, *n* = 41).

**Conclusions:**

Adherence to ambrisentan was good, but its persistence at 1 year was low. The dosages of the drug used were in accordance with the recommendations of the clinical practice guidelines, and it was used in combination therapy, especially with phosphodiesterase 5 inhibitors.

## Graphical abstract

This group of Colombian patients with diagnosis of pulmonary hypertension and were undergoing treatment with ambrisentan is mainly women, approximately 51 years of age. They are often receiving combined therapy with other medications, especially phosphodiesterase 5 inhibitors, at the dosages recommended by clinical practice guidelines. In patients with pulmonary hypertension, the adherence to therapy with ambrisentan was good, but persistence of drug use after 1 year of treatment was low.

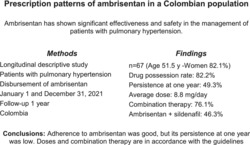

## 1 INTRODUCTION

Pulmonary hypertension is a heterogeneous condition associated with several underlying disorders. It is characterized by marked remodeling of the pulmonary vasculature and by a progressive increase in the pulmonary vascular load, which leads to hypertrophy and remodeling of the right ventricle.^1^ It is defined hemodynamically as a mean pulmonary arterial pressure greater than 20 mmHg at rest measured by right heart catheterization.^1^, ^2^, ^3^, ^4^ According to the underlying mechanism, it is divided into five groups (pulmonary arterial hypertension, due to left heart disease, due to respiratory disease, due to pulmonary artery obstructions, and due to nonestablished/multifactorial mechanisms).^1^, ^2^ Current estimates suggest that pulmonary hypertension has a prevalence of approximately 1% among the world population, increasing to 10% in people over 65 years of age.^5^ Regardless of the underlying disease, the development of pulmonary hypertension is associated with clinical deterioration and a substantially higher risk of mortality.^3^, ^4^, ^5^, ^6^, ^7^

Different drugs with vasodilator properties are approved for lung treatment and have improved the prognosis of patients.^3^, ^4^, ^6^, ^7^, ^8^ Among them is ambrisentan, which is a selective antagonist of endothelin type A receptors approved for the treatment of pulmonary arterial hypertension.^9^, ^10^ Endothelin is a powerful vasoconstrictor with mitogenic, hypertrophic, and proinflammatory properties that is positively regulated in pulmonary hypertensive diseases.^8^ Studies have shown that ambrisentan increases exercise capacity, improves or prevents the deterioration of functional class, and causes favorable changes in cardiopulmonary hemodynamic variables.^11^ Its once‐daily dosing and lower incidence of hepatotoxicity offer potential advantages over other endothelin receptor antagonists.^8^, ^9^

However, patient management recommendations are not totally homogeneous among clinical practice guidelines.^3^, ^4^, ^6^, ^7^ In general, low‐risk patients can be treated initially with monotherapy or combination therapy, while intermediate‐ or high‐risk patients are initially managed with combination therapy involving drugs that have different mechanisms of action.^3^, ^4^, ^6^, ^7^ Among the most recommended combination therapies is the association of an endothelin receptor antagonist with a type 5 phosphodiesterase inhibitor.^3^, ^4^, ^6^, ^7^ In addition to an adequate therapeutic scheme, adherence and persistence to medications also influence the prognosis of patients.^12^ Lack of adherence is a multifactorial problem and may be related to the patient, the health care providers, the disease itself, and the drug and can occur from prescription/dispensing to taking the recommended dosage.^12^

The Colombian Health System offers universal coverage to the entire population through two affiliation regimes: the contributory regime that is paid by workers and employers and the subsidized regime that is responsible for the insurance of all people without the ability to pay and includes a benefit plan that covers some medications used in the management of pulmonary hypertension, including ambrisentan.^10^ Based on the benefits of endothelin receptor antagonists, especially ambrisentan, it is necessary to describe its use, dosage, efficacy in monotherapy or in combination therapy, patients' persistence and adherence, and other pharmacological variables, especially because this information is unknown in Colombia, and it is very limited internationally.^13^, ^14^, ^15^ Therefore, the objective of this study was to determine the prescription patterns of ambrisentan in some cities of Colombia from 2021–2022.

## 2 MATERIALS AND METHODS

### 2.1 Study design and patients

An observational and longitudinal study was carried out to establish the prescription patterns of ambrisentan (Brisen®) in patients with a diagnosis of pulmonary hypertension (all the groups) based on a population database of drug dispensing that collects information from approximately 9.2 million people affiliated with the Colombian Health System and insured by five health insurance companies, corresponding to approximately 30.0% of the active affiliated population of the contributory or payment scheme and 11.0% of the state‐subsidized scheme, which comprises 18.0% of the Colombian population.

Patients were identified from the first disbursement of ambrisentan (Brisen®) between January 1 and December 31, 2021. The first dispensation was considered the index date for each subject. Follow‐up was carried out for 1 year (maximum until December 31, 2022) or until the initiation of another endothelin receptor antagonist (bosentan or macitentan). Patients of any sex and age were selected and attended outpatient medical consultations. Patients who were pregnant and those with a diagnosis of idiopathic pulmonary fibrosis were excluded.

### 2.2 Variables

Information about the consumption of drugs among the affiliated population was systematically obtained by the dispensing company (Audifarma SA), and a database was designed that allowed the following groups of variables to be collected from the patients:

1. Sociodemographic: age, sex, type of affiliation with the health system (contributory or subsidized regime), and city of dispensation. The place of residence was categorized by departments according to the regions of Colombia, considering the classification of the National Administrative Department of Statistics (DANE) of Colombia, as follows: Bogotá‐Cundinamarca region, Caribbean, central region, Eastern region, Pacific region, and Amazon‐Orinoquía region.
2. Pharmacological treatment
  - Ambrisentan:
    - Dose, titration, and duration of therapy. The defined daily dose (DDD) was used as the unit of measurement for the use of drugs, according to the recommendations of the World Health Organization (WHO).
    - Persistence at 1 year: Persistent patients are those who received uninterrupted treatment. A grace period can be established for everyone. If the patient exceeds this predetermined interval, it is considered not persistent.^16^ The grace period used was an interval between dispensations <90 days.^17^
    - Adherence: Adherence was determined with the Medication Possession Ratio (MPR). It measures the percentage of time that a patient has access to medications (MPR = days of supply of medication dispensed/days from the first dispensing to the end of the follow‐up period × 100).^16^ The threshold used to define adherence was an MPR ≥ 80.0%.^16^
  - Other pulmonary vasodilators: Phosphodiesterase type 5 inhibitors (sildenafil or tadalafil), soluble guanylate cyclase stimulants (riociguat), prostacyclin analogs (epoprostenol or iloprost; treprostinil: not available), endothelin receptor antagonists (bosentan or macitentan; sitaxentan: not available), and calcium antagonists (amlodipine, nifedipine, and diltiazem) were included. Patients in monotherapy (only with ambrisentan) and patients in combination therapy (regimens with ambrisentan) were determined.
  - Comedications: These were grouped into the following categories: (a) antidiabetic drugs, (b) antihypertensive and diuretic drugs, (c) lipid‐lowering drugs, (d) antiulcer drugs, (e) antidepressants, (f) anxiolytics and hypnotics, (g) thyroid hormone, (h) antipsychotics, (i) antiepileptics, (j) analgesics and anti‐inflammatories, (k) bronchodilators and/or inhaled corticosteroids, (l) antiplatelet drugs, (m) anticoagulants, (n) micronutrients, and (o) antihistamines, among others.
3. Comorbidities: comorbidities were identified from the main and secondary diagnoses through the codes of the International Classification of Diseases, version 10 (ICD‐10) of the selected patients.
4. Clinical characteristics: the clinical variables of a subgroup of patients undergoing treatment with ambrisentan who had clinical records that were provided by a Colombian Health System insurer were analyzed. The variables of interest were classification of pulmonary hypertension, etiology, risk stratification, functional class according to the WHO, reports of natriuretic peptides, electrocardiogram, echocardiogram, and catheterization of the right heart, hospitalizations during follow‐up, use of home oxygen, use of ambrisentan in approved (Group 1: pulmonary arterial hypertension) and unapproved indications, adverse drug reactions, and tolerability to treatment.

### 2.3 Ethical statement

The protocol was approved by the Bioethics Committee of the Technological University of Pereira in the category of research without risk (Endorsement Code: 13‐130 223), who endorsed the protocol in general and the review of the clinical records of the subgroup of patients. The ethical principles established by the Declaration of Helsinki were respected. Likewise, the patients' insurer provided them with access to clinical and paraclinical information.

### 2.4 Data analysis

The data were analyzed with the statistical package SPSS Statistics, version 26.0 for Windows (IBM, United States). A descriptive analysis was carried out with frequencies and proportions for the qualitative variables and measures of central tendency and dispersion for the quantitative variables by means (standard deviation) and medians (interquartile range). The comparison of quantitative variables was performed using the X^2^ test or Fisher's exact test. A *p* < 0.05 was determined as a level of statistical significance.

## 3 RESULTS

### 3.1 Sociodemographics

Sixty‐seven patients with an initial prescription of ambrisentan (Brisen®) were identified in 10 cities. A total of 82.1% (*n* = 55) were women, and the median age was 51.5 years (interquartile range: 39.8–64.0 years; range: 21.0–83.0 years). A total of 23.9% (*n* = 16) were less than 40 years old, 52.2% (*n* = 35) were between 40 and 64 years old, and 22.4% (*n* = 15) were 65 years or older. Most were in the Bogotá‐Cundinamarca region and were affiliated with the contributory insurance scheme (Table 1).

**TABLE 1:** Sociodemographic variables, comorbidities, and comedications of a group of patients with pulmonary hypertension treated with ambrisentan, Colombia

Variables	Total	
	*n* = 67	%
Women	55	82.1
Age, median (IQR)	51.5 (39.8–64.0)	
Age ≥65 years	15	22.4
Origin	‐	‐
Bogotá‐Cundinamarca Region	48	71.6
Caribbean Region	8	11.9
Central Region	8	11.9
Pacific Region	2	3.0
Eastern‐Orinoquia‐Amazonia Region	1	1.5
Affiliation regime	‐	‐
Contributory	59	88.1
Subsidized	6	9.0
Special	2	3.0
Comorbidities	‐	‐
Arterial hypertension	51	76.1
Chronic obstructive pulmonary disease	19	28.4
Heart failure	14	20.9
Hypothyroidism	14	20.9
Atrial fibrillation	13	19.4
Diabetes mellitus	12	17.9
Ischemic heart disease	10	14.9
Systemic sclerosis	9	13.4
Chronic pain	7	10.4
Obstructive sleep apnea/hypopnea syndrome	5	7.5
Comedications	‐	‐
Antihypertensives and diuretics	36	53.7
Analgesics and anti‐inflammatories	35	52.2
Micronutrients	23	34.3
Anticoagulants	17	25.4
Antihistamines	15	22.4
Hypolipidemics	15	22.4
Inhaled bronchodilators and corticosteroids	14	20.9
Thyroid hormone	14	20.9
Antiplatelet agents	13	19.4
Antidepressants	12	17.9

Abbreviation: IQR, interquartile range.

### 3.2 Ambrisentan

The median adherence of all patients was 82.2% (interquartile range: 65.0–96.8%), and 62.7% (*n* = 42) had an MPR ≥ 80.0%. A total of 49.3% (*n* = 33) were persistent at 1 year. Of these patients, 30.3% (*n* = 10/33) had continuous treatment without interruptions during the 12 months of follow‐up. A total of 20.9% (*n* = 14) received the drug ≤90 days, 22.4% (*n* = 15) between 91 and 180 days, 19.4% (*n* = 13) between 181 and 270 days, and 37.3% (*n* = 25) for more than 270 days. A total of 38.8% (*n* = 26) were new patients to ambrisentan during the study period, and 76.1% (*n* = 51) received combination therapy, especially with phosphodiesterase type 5 inhibitors (*n* = 41; 61.2%). Fourteen different treatment schemes were found, and 88.1% (*n* = 59) of the patients were managed with ambrisentan + sildenafil (*n* = 31; 46.3%), ambrisentan alone (*n* = 16; 23.9%), ambrisentan + tadalafil (*n* = 6, 9.0%), ambrisentan + amlodipine (*n* = 2, 3.0%), ambrisentan + riociguat (*n* = 2, 3.0%), or ambrisentan + sildenafil + amlodipine (*n* = 2, 3.0%). Table 2 shows the pattern of use of ambrisentan, the frequency of use, prescribed dosages, and the distribution by sex and age.

**TABLE 2:** Pattern of use of ambrisentan in monotherapy and combined therapy, frequency of use, prescribed dose, and distribution by sex and age, in a group of patients with pulmonary hypertension, Colombia

Drugs	*n* = 67	%	Prescribed dose (mg/day)				Sex		Age	
			Mean ± SD	Median (IQR)	Mode	nDDD^a^	F (%)	M (%)	Mean ± SD	Median (IQR)
Ambrisentan	67	100.0	8.8 ± 5.0	8.3 (5.0–10.9)	5.0	1.17	82.1	17.9	51.9 ± 15.5	51.5 (39.8–64.0)
Combined	51	76.1	9.2 ± 5.4	8.6 (5.0–11.0)	5.0	1.23	80.4	19.6	49.4 ± 15.7	49.5 (34.8–63.0)
Monotherapy	16	23.9	7.6 ± 2.8	6.6 (5.0–10.0)	5.0	1.01	87.5	12.5	59.8 ± 12.3	63.5 (50.8–67.8)
Others	‐	‐	‐	‐	‐	‐	‐	‐	‐	‐
Sildenafil	34	50.7	98.4 ± 55.2	75.0 (60.0–126.7)	60.0	1.96	79.4	20.6	46.8 ± 13.7	47.0 (34.0–56.0)
Tadalafil	7	10.4	38.9 ± 37.5	25.3 (18.7–40.0)	40.0	3.89	85.7	14.3	55.0 ± 17.8	55.0 (38.0–66.0)
Riociguat	5	7.5	6.6 ± 1.0	7.0 (5.9–7.1)	7.0	1.46	80.0	20.0	55.6 ± 20.2	52.0 (36.5–76.5)
Nifedipine	4	6.0	55.3 ± 27.8	50.5 (31.5–83.8)	30.0	1.84	75.0	25.0	59.3 ± 6.4	63.0 (57.5–63.0)
Amlodipine	3	4.5	12.6 ± 6.8	14.4 (9.7–16.4)	5.0	2.52	66.7	33.3	57.7 ± 21.2	64.0 (49.0–69.5)
Iloprost	3	4.5	0.10 ± 0.03	0.12 (0.09–0.12)	0.12	0.66	66.7	33.3	40.7 ± 13.6	39.0 (33.5–47.0)
Trepostinil	1	1.5	6.7	6.7	6.7	1.55	100.0	0.0	31.0	31.0
Diltiazem	1	1.5	112.5	112.5	112.5	0.46	100.0	0.0	70.0	70.0

Abbreviations: F, female; IQR, interquartile range; M, male; SD, standard deviation.

^a^ Proportion between the daily dose received and the defined daily dose.

A total of 7.5% (*n* = 5) of the patients changed their pharmaceutical form. A total of 6.0% (*n* = 4) went from 5 to 10 mg tablets, and only one (1.5%) went from 10 to 5 mg. The median time from the index date to the change was 154.0 days (interquartile range: 110.5–224.0 days). A total of 91.0% (*n* = 61) of the patients received only ambrisentan (Brisen®) during follow‐up, while 9.0% (*n* = 6) received ambrisentan (Brisen®) and ambrisentan from another commercial reference.

A total of 76.1% (*n* = 51) received medications for pulmonary hypertension in the year prior to the index date, mainly sildenafil (*n* = 34, 50.7%), bosentan (*n* = 10, 14.9%), tadalafil (*n* = 7, 10.4%), nifedipine (*n* = 6, 9.0%), amlodipine (*n* = 5, 7.5%), and riociguat (*n* = 5, 7.5%).

### 3.3 Comorbidities and comedications

A total of 94.0% (*n* = 63) of the patients had some comorbidity. Most of the patients had arterial hypertension, and the most frequently encountered comedications were antihypertensives and diuretics (see Table 1).

### 3.4 Clinical characteristics

A total of 26.9% (*n* = 18) of the patients had access to some of the clinical data, mainly through the electronic medical records in the general medicine department (*n* = 17; 94.4%) and internal medicine department (*n* = 10; 55.6%). Most of the patients were classified in Group 1; in the etiology, the cases with pulmonary thromboembolism predominated, and they had a WHO Functional Class I (Table 3). Ambrisentan was used mainly in approved indications in 55.6% (*n* = 10) of the cases, but it was also possible to identify its use in nonapproved indications (*n* = 5; 27.8%), and one patient did not tolerate therapy.

**TABLE 3:** Clinical variables of a subgroup of patients with pulmonary hypertension treated with ambrisentan, affiliated with a single insurer in Colombia

Variables	Total	
	*n* = 18	%
Classification	‐	‐
Group 1	6	33.3
Group 4	4	22.2
Groups 1 and 3	4	22.2
Group 3	1	5.6
Unknown	3	16.7
Etiology	‐	‐
Chronic thromboembolism	4	22.2
Idiopathic	4	22.2
Atrial septal defect	2	11.1
Ventricular septal defect	1	5.6
Valve disease (unspecified)	1	5.6
Scleroderma	1	5.6
Systemic lupus erythematosus	1	5.6
Eisenmenger syndrome–Cor pulmonale	1	5.6
Unknown	3	16.7
WHO Functional Class	‐	‐
I	11	61.1
II	3	16.7
III	1	5.6
Unknown	3	16.7
Echocardiogram (*n* = 11)^a^	‐	‐
LVEF (%), median (IQR); (*n* = 9)^a^	60.0 (58.5–69.5)	
PSAP (mmHg), median (IQR); (*n* = 7)^a^	60.0 (42.0–85.0)	
Hospitalizations	3	16.7
Desaturation (unspecified)	1	5.6
Heart failure with decompensation	1	5.6
Community‐acquired pneumonia	1	5.6
Home oxygen	6	33.3

Abbreviations: IQR, interquartile range; LVEF, left ventricular ejection fraction; PSAP, pulmonary artery systolic pressure; WHO, World Health Organization.

^a^ Patients with clinical information available.

### 3.5 Exploratory bivariate analysis

In an exploratory bivariate analysis, patients aged between 40 and 64 years (OR: 3.2; 95%CI: 1.18–8.79; *p* = 0.020), those undergoing treatment with ambrisentan in combination with other medications (OR: 6.19; 95%CI: 1.56–24.45; *p* = 0.005), and those who had arterial hypertension as a comorbidity (OR: 3.95; 95%CI: 1.12–13.94; *p* = 0.026) were more persistent per year.

## 4 DISCUSSION

This study allowed us to identify the pattern of use of ambrisentan in a group of patients with pulmonary hypertension as evidence of the use of drugs in the real world. Ambrisentan was used mainly in women under 65 years of age and with pulmonary arterial hypertension (Group 1). Most were in combined management, especially with phosphodiesterase type 5 inhibitors, showing adequate adherence but with low persistence at 1 year. Knowing the patterns of use of medicines promotes their appropriate use and reduces their abuse or misuse.^18^ Irrational drug use is a major problem worldwide.^19^ The WHO estimates that more than half of all medicines are improperly prescribed, dispensed, or sold and that half of all patients do not take them correctly.^19^

The median age of the patients in this study was similar to that found in some studies in Asia (47.0–50.8 years)^20^, ^21^ and Europe (49.6–52.0 years),^22^, ^23^, ^24^ but in other reports from Europe (61.0–61.6 years),^13^, ^14^, ^25^ North America (62.7–68.1 years)^17^, ^26^, ^27^, ^28^ and Oceania (60.0 years),^29^ patients were older,^13^, ^14^, ^17^, ^25^, ^26^, ^27^, ^28^, ^29^ and there was a predominance of women (82.1% vs. 61.0–79.0%).^13^, ^14^, ^15^, ^17^, ^20^, ^21^, ^22^, ^23^, ^24^, ^25^, ^26^, ^27^, ^28^, ^29^, ^30^, ^31^ Arterial hypertension was the most prevalent comorbidity in this report, which is consistent with the literature.^17^, ^20^, ^26^, ^27^, ^28^, ^29^, ^30^, ^31^ Pulmonary arterial hypertension (Group 1) was the most prevalent, in line with other studies with real‐world evidence,^20^, ^22^, ^23^, ^25^ and most of the patients had WHO Functional Class I; this differs from other reports in which the patients presented a more deteriorated functional class,^15^, ^20^, ^22^, ^23^, ^25^, ^29^ but the proportion of cases with home oxygen use was similar (33.3% vs. 21.6–30.2%).^24^, ^28^

The dosages of ambrisentan used were in accordance with the recommendations of the clinical practice guidelines^3^, ^4^, ^6^, ^7^ and regulatory agencies.^9^, ^10^ The most commonly used dosage was 5 mg/day, which is consistent with that reported in other investigations.^14^, ^15^ Combination therapy prevailed in this study, most often with phosphodiesterase type 5 inhibitors, which is consistent with studies conducted in Sweden^13^ and United Kingdom^14^ on patterns of use of ambrisentan^13^, ^14^ and is in line with the recommendations of the clinical practice guidelines.^3^, ^4^, ^6^, ^7^ In other studies on the pharmacological management of pulmonary hypertension, it has been shown that monotherapy predominates in most cases (51.0–95.1%).^17^, ^22^, ^26^, ^27^, ^28^, ^29^ This can be explained by the methodological differences of the studies, since some of them include only incident cases and others include both incidence and prevalence; it will also depend on the severity of the pathology, the level of risk, the tolerability of the drugs, the moment in which the research was carried out, and the recommendations in force at the time of the study.^3^, ^4^, ^6^, ^7^, ^17^, ^22^, ^26^, ^27^, ^28^, ^29^

Adherence to ambrisentan in this group of patients was high and is in line with what has been described in other studies.^13^, ^17^, ^28^, ^30^, ^32^ In Sweden, Kjellström et al.,^13^ using the same methodology (MPR), documented that adherence to ambrisentan was 87%. Other reports have grouped endothelin receptor antagonists and have found adhesions ranging from 75.6% to 95.0%.^17^, ^28^, ^30^, ^32^ However, persistence per year in this report was not high, which is consistent with the literature.^14^, ^15^, ^17^, ^28^ In the United Kingdom, 54% of patients continued to receive ambrisentan for an average of 3.2 years.^14^ In the United States, 62.0% of patients continued to be treated with endothelin receptor antagonists,^17^, ^28^ and in a multicenter study involving 15 countries in Europe, Canada, and Australia, 53.0% continued to receive this therapeutic group during follow‐up.^15^ The explanations for the interruption of therapy can be multicausal, including the lack of perception of the benefit of the treatment, the presence of adverse drug reactions, lack of continuity in medical care, inadequate coverage, and high drug costs.^12^ The importance of having high adherence and persistence is that the loss of continuity of management leads to a deterioration in the prognosis of patients.^12^

A systematic review and meta‐analysis of 14 studies (*n* = 14 861) that involved patients with pulmonary hypertension and undergoing pharmacological treatment showed that the proportion of adherent patients was 60.9% and the proportion of those who discontinued medications was 42.3%.^33^ The influencing factors were the frequency of administration, the duration of treatment, the cost, and the appearance of adverse drug reactions.^33^ On the other hand, in this report, it was found that patients with intermediate ages, with an additional diagnosis of arterial hypertension and on combined pharmacological treatment for pulmonary hypertension, were more persistent. Older adults may be less adherent and persistent to pharmacological management related to cognitive impairment, its multimorbidities, and polypharmacy.^34^ It is possible that patients who are being treated for arterial hypertension better tolerate the potential hypotension that some of the medications used in the management of pulmonary hypertension can produce and therefore discontinue their treatment less.^9^, ^35^ Finally, patients on combined therapy were more persistent because they could have had more severe pulmonary hypertension, which responded favorably with pharmacological management, which could improve functional class and increase the distance of the 6 min walk test.^36^ However, it should be taken into account that the combination of medications increases the risk of adverse drug reactions.^36^, ^37^ Because several factors impact treatment adherence and persistence, comprehensive interventions are needed to improve patient outcomes.^33^

Some limitations should be considered when interpreting the results. The total population of patients undergoing drug treatment with ambrisentan is low, and the study participants came predominantly from the Bogotá‐Cundinamarca region, so it is possible that the results cannot be generalized to all regions of the country. In addition, it was possible to access the clinical records of less than a third of the cases, and we did not find reports of natriuretic peptides, electrocardiograms, or hemodynamic data of right heart catheterizations. It was also not possible to determine the risk stratification of the patients or mortality during follow‐up.

## 5 CONCLUSIONS

The results of this study indicate that this group of Colombian patients affiliated with the health system who had a diagnosis of pulmonary hypertension and were undergoing treatment with ambrisentan (Brisen®) are mainly women, approximately 51 years of age. They are often receiving combined therapy with other medications, especially phosphodiesterase 5 inhibitors, at the dosages recommended by clinical practice guidelines. The most common comorbidity was arterial hypertension. It was also possible to identify that adherence to therapy with ambrisentan was good, but persistence of drug use after 1 year of treatment was low. Strategies that seek to reduce interruptions to pharmacological management should be strengthened to improve the prognosis of patients.

## AUTHOR CONTRIBUTIONS

**Luis Fernando Valladales‐Restrepo**: Conceptualization; methodology; formal analysis; investigation; data curation; writing of the original draft. **Andrés Gaviria‐Mendoza**: Formal analysis; investigation; data curation. **Manuel Enrique Machado‐Duque**: Formal analysis; investigation; data curation. **Álvaro Vallejos‐Narváez**: Methodology; resources. **Jorge Enrique Machado‐Alba**: Methodology; validation; formal analysis; resources; writing; review and editing; supervision.

## CONFLICT OF INTEREST STATEMENT

LFVR, AGM, MEMD, and JEMA have a contractual relationship with Audifarma SA, the entity that received the research grant. AVN works at Megalabs Colombia.

## ETHICS STATEMENT

The protocol was approved by the Bioethics Committee of the Technological University of Pereira in the category of research without risk (Endorsement Code: 13‐130 223), who endorsed the protocol in general and the review of the clinical records of the subgroup of patients. The ethical principles established by the Declaration of Helsinki were respected. Likewise, the patients' insurer provided them with access to clinical and paraclinical information.

## Data Availability

The data that support the findings of this study are openly available in protocols.io (at https://www.protocols.io/private/14ACDB935C7A11EE9B570A58A9FEAC02).
